# Key regulators in prostate cancer identified by co-expression module analysis

**DOI:** 10.1186/1471-2164-15-1015

**Published:** 2014-11-24

**Authors:** Junfeng Jiang, Peilin Jia, Zhongming Zhao, Bairong Shen

**Affiliations:** Center for Systems Biology, Soochow University, Jiangsu, China; Department of Biomedical Informatics, Vanderbilt University School of Medicine, Nashville, TN 37203 USA; Center for Quantitative Sciences, Vanderbilt University Medical Center, Nashville, TN 37232 USA; Department of Psychiatry, Vanderbilt University School of Medicine, Nashville, TN 37212 USA; Department of Cancer Biology, Vanderbilt University School of Medicine, Nashville, TN 37232 USA

**Keywords:** Prostate cancer, Co-expression, Gene Ontology, Module, Transcription factor, MicroRNA

## Abstract

**Background:**

Prostate cancer (PrCa) is the most commonly diagnosed cancer in men in the world. Despite the fact that a large number of its genes have been investigated, its etiology remains poorly understood. Furthermore, most PrCa candidate genes have not been rigorously replicated, and the methods by which they biologically function in PrCa remain largely unknown.

**Results:**

Aiming to identify key players in the complex prostate cancer system, we reconstructed PrCa co-expressed modules within functional gene sets defined by the Gene Ontology (GO) annotation (biological process, GO_BP). We primarily identified 118 GO_BP terms that were well-preserved between two independent gene expression datasets and a consequent 55 conserved co-expression modules within them. Five modules were then found to be significantly enriched with PrCa candidate genes collected from expression Quantitative Trait Loci (eQTL), somatic copy number alteration (SCNA), somatic mutation data, or prognostic analyses. Specifically, two transcription factors (TFs) (NFAT and SP1) and three microRNAs (hsa-miR-19a, hsa-miR-15a, and hsa-miR-200b) regulating these five candidate modules were found to be critical to the development of PrCa.

**Conclusions:**

Collectively, our results indicated that genes with similar functions may play important roles in disease through co-expression, and modules with different functions could be regulated by similar genetic components, such as TFs and microRNAs, in a synergistic manner.

**Electronic supplementary material:**

The online version of this article (doi:10.1186/1471-2164-15-1015) contains supplementary material, which is available to authorized users.

## Background

Prostate cancer (PrCa) is the sixth leading cause of cancer-related deaths of men in the world [1] and the second leading cause in the United States [2]. Due to the high risk of metastasis, it has become fundamentally important to uncover the underlying mechanisms of PrCa. Factors such as age, ethnicity, family history, heritability, diet, lifestyle, environment, and androgens have long been recognized as contributors to the risk of PrCa [3–5]. As demonstrated by twin studies, PrCa’s genetic component is estimated to be as high as 42-57% [6, 7].

To elucidate the underlying pathophysiology and molecular mechanisms of PrCa, numerous genetic and genomic studies have been conducted, including gene expression profiling [8–12], expression Quantitative Trait Loci (eQTL) mapping [13–15], somatic copy number alteration (SCNA) identification [16], gene mutation detection [17], prognostic gene discovery [18], microRNA (miRNA) expression profiling [14, 19], and transcription factor (TF) enrichment [20], among others. The gene expressions profiled by microarray technology have been a major strategy to detect mRNA abundance. Traditional, single, and gene-based strategies have been widely applied for gene expression analyses, but they suffered from limitations, such as multiple testing burdens [21], small numbers of differentially expressed genes [22], lack of interactions/regulations among genes [23], or low replication rates [24].

Alternatively, gene co-expression module analysis attempts to study combined effects by identifying groups of genes that are coordinately expressed [21, 25–27]. For instance, Horvath and colleagues have developed a widely used algorithm, the Weighed Gene Co-expression Network Analysis (WGCNA) [28], to search for co-expression modules. The R package WGCNA implements a suite of tools for network construction, module detection, module significance examination, module preservation computation, and hub gene query, among many others [29–31].

**Figure 1:**
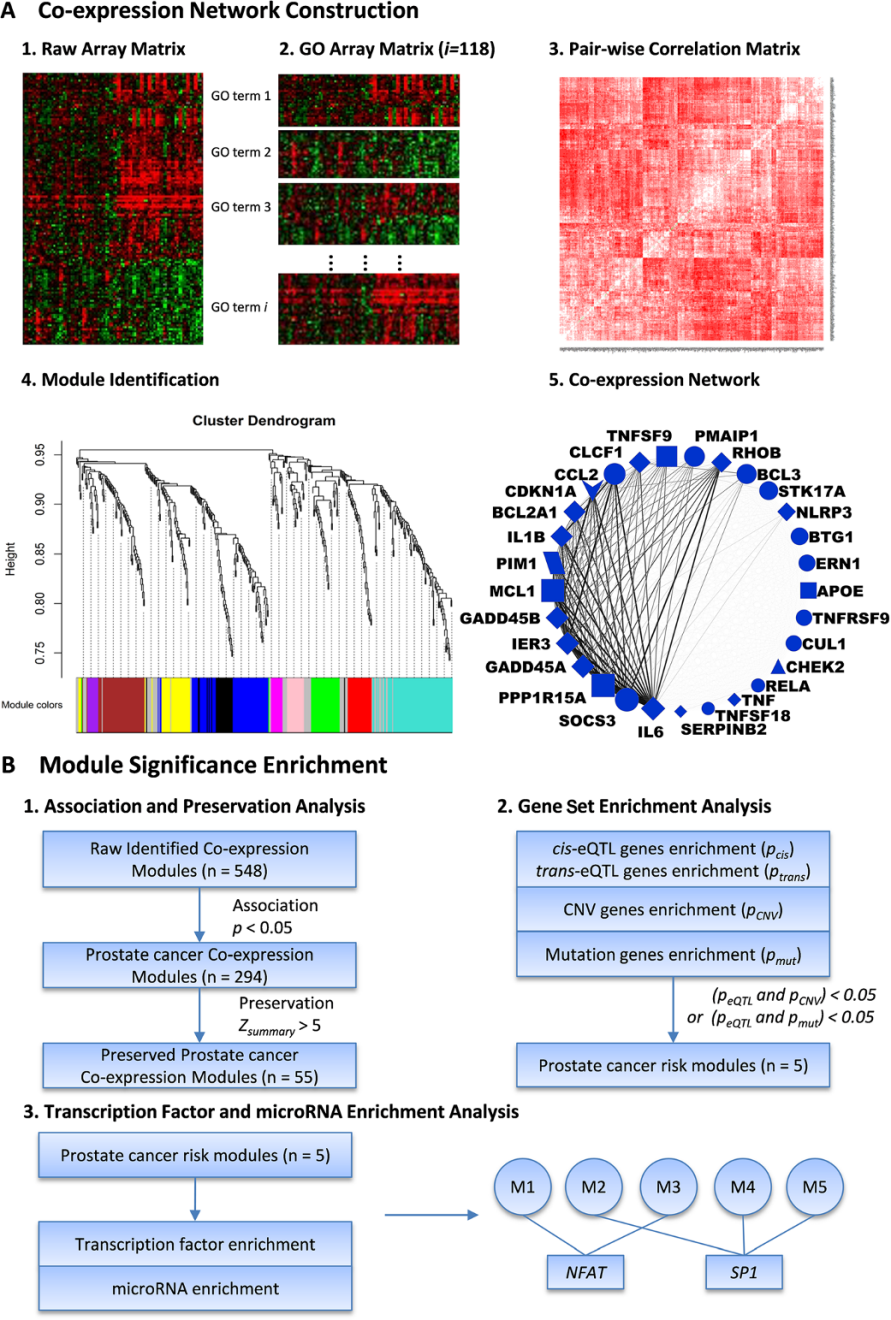
**Overview of workflow. (A)** GO-based gene co-expression network construction. It has five steps: (1) Raw microarray data processing and analysis. (2) GO term expression data matrix-building. (3) Pairwise correlation analysis of genes in GO term across different samples. (4) Expression profile clustering and module identification using WGCNA. (5) Visualization of co-expression modules by Cytoscape. **(B)** Framework of module significance analyses. The details are provided in Methods.

To our knowledge, no co-expression module has been constructed for the identification of key regulators in PrCa until now. Moreover, traditional co-expression studies that start from whole human genes on a chip (or top differentially expressed genes) often result in very large modules (e.g., >1000 genes). Although functional assessments, such as GO enrichment, the functional gene/SNP enrichment test, and hub gene analysis could help to explore the functions of modules, such interpretation typically results in noisy results (e.g., a lot list of GO terms or genes). In this study, we developed a framework for gene co-expression module construction in PrCa using the WGCNA approach and augmented by Gene Ontology [32] biological process (GO_BP) annotations. We argued that although GO_BP terms are broadly defined for each functional group, there may be subsets of genes in a biological process (GO_BP term) that are coordinately expressed, e.g., in a disease-associated fashion. For example, different co-expression modules may underlie different diseases, although they all execute the same biological functions as defined by GO_BP terms. To this end, we developed a systematic framework (Figure 1) to search for co-expression modules within each GO_BP term and demonstrated it in PrCa. We primarily found 118 preserved GO_BP terms in two PrCa datasets and constructed 55 co-expression modules. We then assessed these modules for their enrichment of PrCa candidate genes collected by eQTLs, SCNA, somatic mutation data, or prognostic studies using the hypergeometric test. As a result, 5 modules were identified as significantly associated with PrCa, and several TFs and miRNAs were found to be potential key regulators of these candidate modules.

## Methods

### Processing and analysis of microarray gene expression data

Two microarray gene expression datasets were downloaded from the National Center for Biotechnology Information (NCBI) Gene Expression Omnibus (GEO) database (http://www.ncbi.nlm.nih.gov/geo/). To ensure the data quality, we searched for studies that have a well-defined phenotypic description for PrCa, have a sample size around 100 or more, and have preferably been measured using similar platforms in order to obtain a high proportion of overlapping genes. We included both case and control samples to determine disease-specific signals, as similarly done in Chen *et al*. [21]. Two datasets that fulfilled these criteria were downloaded for our further analyses (Table 1). The first dataset (GEO accession ID: GSE17951 [33]), which was used as the training dataset in our work, was generated using the Affymetrix U133Plus2 array on prostate tissue samples from 109 cancer patients and 45 control samples [33] (Table 1). The second dataset (GEO accession ID: GSE6956 [10]), which was used as the testing dataset, was collected using the Affymetrix Human Genome U133A 2.0 array for 69 fresh-frozen prostate tumors and 20 control samples from surrounding normal prostate tissue [10]. For each dataset, we performed the following quality control steps. First, outlier samples were detected and removed. We calculated the inter-array correlation (IAC) based on Pearson’s correlation coefficient for tumor and control samples, respectively. We excluded the samples with low mean IAC and/or those that emerged as a clear outlier by the hierarchical clustering approach [34]. Second, samples were quantile-normalized after log_2_ transformation [35]. Probes with missing expression values in more than 30% samples were removed from further analyses. For each gene, we selected the probe with the highest intensity to represent the expression level of the gene. This resulted in 21,049 genes involved in 82 tumor and 40 control samples in the training dataset and 13,211 genes in 60 tumor and 19 normal samples in the testing dataset, with 13,211 genes shared by the two datasets.

**Table 1:** **Summarization of PrCa microarray gene expression datasets used in the study**

GEO accession ID	Type	Before QC^a^		After QC		# Genes
		# Tumors	# Controls	# Tumors	# Controls	
GSE17951	Training	109	45	82	40	21049
GSE6956	Testing	69	20	60	19	13211

^a^QC: quality control.

### Highly-preserved GO_BP terms

The GO database provides three annotation categories (domains): Molecular Function (MF), Biological Process (BP), and Cellular Component (CC). In this study, we focused on the BP category, based on the notion that genes that participate in the same biological processes tend to be expressed coordinately [36]. We downloaded the GO_BP gene sets from the Molecular Signatures Database (MSigDB) [37], including 825 GO_BP terms in the C5 category. To avoid too broadly or too narrowly defined GO_BP terms, we only kept the terms with 50 to 500 measured genes. As a result, 226 GO_BP terms were eligible to build the gene expression matrices for the following analysis.

Before the construction of the modules, we first evaluated the importance of each term associated with PrCa by calculating a preservation score. The preservation score aims to assess the level of preservation between the training and testing datasets for a gene set (i.e., a GO_BP term) and is typically measured based on both density and connectivity patterns among the genes [29]. The parameter *Z*_summary_[29] implemented in WGCNA [28] was employed to compute the preservation score. In general, a value of *Z*_summary_ <2 indicates no evidence of preservation between the training and the testing datasets, 2 < *Z*_summary_ <10 implies weak to moderate evidence, and *Z*_summary_ >10 indicates strong evidence. At this stage, we aim to perform pre-selection of GO_BP terms that are suitable for the following co-expression clustering analysis. To this end, we chose a moderate threshold of preservation, *Z*_summary_ = 5 [29], to select GO_BP terms and denote them as preserved GO_BP terms in both training and testing datasets.

### Application of WGCNA in PrCa

Given that genes within a GO_BP term are well-defined with similar biological functions, we asked whether they tend to co-express in a specific disease. Thus, we performed gene co-expression analysis for each preserved GO_BP term instead of all genes on the chip. We took the gene expression matrix for each GO_BP term as the input and applied WGCNA to detect co-expression modules. Gene co-expression correlation was measured by Pearson’s correlation coefficients. In this step, we built a pairwise co-expression matrix. This GO_BP term-based matrix was then utilized to construct an initial gene co-expression network by the *blockwiseModules* function in WGCNA. Notably, the initial co-expression network constructed and based only on Pearson’s correlation coefficients was not always a scale-free network. Rather, to obtain a scale-free network, a weighted adjacency matrix needs to be constructed using a selected power determined through a soft-thresholding approach in WGCNA.

Co-expression modules were then defined by a robust dynamic hierarchical tree cut algorithm using the measurement of dissimilarity (i.e., 1-topological overlap matrix) [26, 38]. To ensure a suitable number of genes for next-step analysis, we set the minimum module size as 10. The adjacent modules were merged based on the parameter of cutHeight, i.e., modules with a minimum cutHeight at 0.25 were merged. Principle component analysis (PCA) of the expression matrix for each module was then performed. We denoted the first principal component (PC) as the module eigengene and used it to represent the overall expression profile of the module [39]. For each gene, we computed a module membership (kME) based on the correlation between the gene expression and the module eigengene. Those genes with a lower membership (kME ≤0.3) were removed from the module and assigned to the grey module.

To validate whether the identified modules were associated with PrCa, we conducted a two-step evaluation procedure. First, for each module, we adopted the module eigengene to assess its trait association (denoted as *p*_cor_) based on Pearson’s correlation coefficients. We used the false positive rate (FDR) for multiple testing correction [40]. Second, for PrCa-associated modules, we further evaluated the module preservation in the testing dataset. Since the size of our identified module was generally less than 100, we defined a module to be preserved if it has a *Z*_summary_ (module) >5.

### Enrichment test

Four types of large-scale, PrCa-associated genetic/genomic data, eQTL genes, recurrent SCNAs, somatic mutations, and prognostic genes were collected for the enrichment test of the identified modules. The eQTL genes were collected from the online eQTL database, SeeQTL (http://www.bios.unc.edu/research/genomic_software/seeQTL/), which re-analyzed nine independent HapMap studies in lymphoblastoid cell lines (LCLs); performed a consensus meta-analysis with comprehensive quality control, population stratification control, and FDR control; and provided the q-value as the significance measurement [41–47]. We retrieved a total of 8652 genes regulated by *cis-*eQL (7071 genes) or *trans-*eQTL (4140 genes) from the SeeQTL database. Here, *cis*-eQTL represents the cases where the regulated genes are located within 1 Mb of the regulatory SNP, while *trans*-eQTL indicates associations for more distant eQTL. We denoted them as *cis-*eQTL and *trans-*eQTL gene sets, respectively.

A list of PrCa susceptibility genes located in SCNA regions was downloaded from the cBio data portal [48]. These SCNA regions were obtained using GISTIC2 (q-value <0.1) [49], based on The Cancer Genome Atlas (TCGA) prostate adenocarcinoma data (https://tcga-data.nci.nih.gov/tcga/). In sum, we retrieved 567 unique genes and denoted them as the SCNA gene set.

Genes with somatic mutations were collected from two sources. First, we manually collected 47 mutated genes from the Human Prostate Gene Database (PGDB) [17]. Second, 107 significantly mutated genes identified in the TCGA prostate adenocarcinoma (https://tcga-data.nci.nih.gov/tcga/) samples were retrieved from the cBio portal. As a result, we obtained 149 unique genes and denoted them as the mutant gene set.

We downloaded the RNASeqV2 and clinical data for prostate adenocarcinoma from the TCGA portal (https://tcga-data.nci.nih.gov/tcga/). The Univariate Cox model was applied to define the prognostic genes [18]. FDR was applied for multiple testing correction of the raw Wald *p* values. Finally, we obtained 737 genes with FDR < 0.05.

Gene set enrichment analysis for PrCa-associated modules was performed using the hypergeometric test. Multiple testing correction was controlled by the FDR method. The module was taken as a candidate if it significantly enriched with any of the two gene sets among eQTL, SCNA, mutation, and prognostic genes with FDR < 0.05. We further performed enrichment tests of the candidate module genes with TF and miRNA using the online tool WebGestalt [50]. The resultant *p* values were corrected for multiple testing using the FDR method. We set the significance level for FDR at 0.01 and the minimum number of genes for a category at two. For simplification, the top 5 enriched TFs or miRNAs were collected for further analyses.

## Results

### The identified GO_BP-based co-expression modules

GO_BP terms categorize genes that function in the same or similar biological processes. Hence, genes in the same GO_BP term could be expected to have coordinated expression patterns. In our study, among the 226 GO_BP terms that fulfilled our query criteria (size between 50 and 500), 118 had a preservation score of *Z*_summary_ (GO_BP) >5 and were considered well preserved between the training and the testing datasets. For each of these 118 GO_BP terms, we built a weighted co-expression network using the entire training dataset of 82 prostate tumor samples and 40 control samples (see Methods). Highly co-expressed genes within a term were then clustered into modules, each labeled with a specific color (Figure 1A4). This resulted in 548 modules in total. To examine their association with prostate cancer, we calculated the correlation between the module expression profiles (represented by the module eigengene) and the PrCa disease status (represented by a vector of 1 for case and 0 for control). With FDR <0.05, we identified 294 of the 548 modules that showed statistically significant association with PrCa in the training dataset. To further evaluate the association, we calculated the preservation score, *Z*_summary_, for each single module against the testing dataset. 55 of the 294 modules were preserved with *Z*_summary_ (module) >5. As shown in Additional file 1: Table S1, these 55 modules belong to 35 GO_BP terms. Many of these terms have been reported to be associated with cancer development, such as “biosynthetic process” [18], “cell-cell signaling” [51, 52], “inflammatory response” [53], “response to stress” [54], “post translational protein modification” [55], “immune system process” [56], “phosphorylation” [57], “regulation of apoptosis” [58], and “regulation of cell proliferation” [59] in many cancer types, including PrCa. Although expected, these results confirmed that the identified modules are important to PrCa, and the method for the analysis is reasonable. In addition, the successful detection indicates that there are indeed subsets of genes within each single GO_BP term that are co-expressed and associated with PrCa. This further verified the rationale of our procedure to examine co-expression patterns in each GO_BP term, rather than in the whole gene set on chip, and proved its ability to discover disease-associated genes and modules.

### Characterization of the identified PrCa associated modules

Our enrichment test of the 55 preserved PrCa-associated modules showed that six modules were significantly enriched with PrCa candidate genes, such as eQTL, SCNA, or mutant genes (FDR adjusted *p* < 0.05) (Additional file 1: Table S1). Their functions are annotated as “response to stress (labeled in green in Figure 1A4)” (*p*_*cis*-eQTL_ = 0.017, *p*_*trans*-eQTL_ = 3.16 × 10^−3^, *p*_mutation_ = 1.37 × 10^−3^), “cellular localization (turquoise)” (*p*_*cis*-eQTL_ = 7.30 × 10^−3^, *p*_SCNA_ = 0.024, *p*_prog_ =1.62 × 10^−3^), “protein localization (brown)” (*p*_*cis*-eQTL_ = 0.034, *p*_SCNA_ = 0.039, *p*_mutation_ = 6.84 × 10^−4^), “regulation of apoptosis (green)” (*p*_*cis*-eQTL_ = 8.93 × 10^−3^, *p*_SCNA_ = 0.040), “regulation of apoptosis (red)” (*p*_*cis*-eQTL_ = 0.027, *p*_*trans*-eQTL_ = 6.59 × 10^−3^, *p*_mutation_ = 6.39 × 10^−5^), and “apoptosis go (black)” (*p*_*cis*-eQTL_ = 8.93 × 10^−3^, *p*_*trans*-eQTL_ = 1.08 × 10^−3^, *p*_mutation_ = 6.41 × 10^−5^). We calculated the pairwise similarities between the six modules using Fisher’s exact test. As a result, “regulation of apoptosis (red)” and “apoptosis go (black)” were found with a large proportion of overlapping genes (*p* = 3. 7 × 10^−41^). We therefore merged these two modules and referred to the resultant module as M1. Other modules were listed as M2-M5, as summarized in Table 2.

**Table 2:** **Overview of enrichment analyses of the five identified PrCa modules**

Module ID					***cis***-eQTL		***trans-***eQTL		SCNA		Somatic mutation		Prognostic genes	
	Description	***p***_cor_^a^	Size	***Z***_***summary***_	# genes	***p***_***cis***-eQTL_^a^	# genes	***p***_***trans***-eQTL_^a^	# genes	***p***_SCNA_^a^	# genes	***p***_mutation_^a^	# genes	***p***_prog_^a^
M1	Regulation of apoptosis (red)	6.23 × 10^−8^	29	10.0	17	3.66 × 10^−3^	13	1.01 × 10^−3^	NA	NA	3	1.99 × 10^−4^	NA	NA
	Apoptosis go (black)													
M2	Regulation of apoptosis (green)	1.51 × 10^−2^	26	6.1	15	8.93 × 10^−3^	7	0.11	2	0.040	NA	NA	2	0.081
M3	Response to stress (green)	4.18 × 10^−11^	37	8.3	19	0.017	14	3.16 × 10^−3^	NA	NA	2	1.37 × 10^−3^	NA	NA
M4	Cellular localization (turquoise)	3.27 × 10^−9^	70	5.7	35	7.30 × 10^−3^	9	0.85	6	0.024	NA	NA	10	1.62 × 10^−3^
M5	Protein localization (brown)	8.04 × 10^−10^	24	5.2	13	0.034	5	0.29	2	0.039	2	6.84 × 10^−4^	NA	NA

^a^ *p* values were adjusted by FDR method.

**Figure 2:**
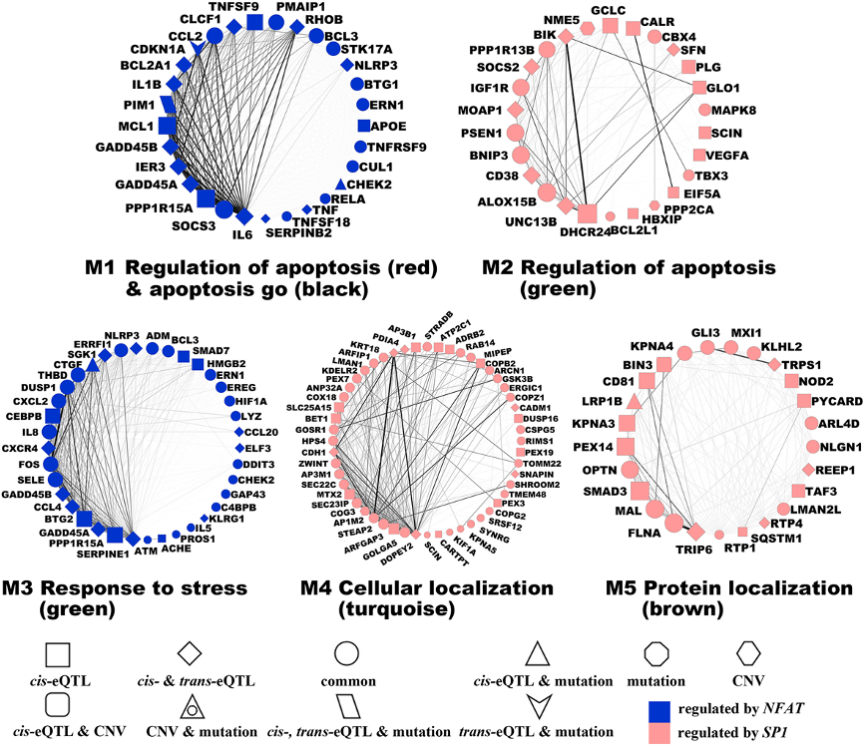
**Visualization of the five identified PrCa-associated modules.** To better describe the modules, we ranked nodes by their module membership (kME) values, and the edge widths are proportional to their correlations. Legends are listed at the bottom for clarity.

For these identified PrCa-associated modules, we recalculated the Module Membership (kME) of each gene by its correlation with the module eigengene (Additional file 2: Table S2). In particular, we presented the five modules in Figure 2, in which nodes were ranked by their kME values and edge thickness reflected the correlations’ coefficients. Node shapes represented different gene functions, as shown in Additional file 2: Table S2. Genes with higher kME values were highly interconnected in modules M1, M3, and M4, indicating a strong co-expression pattern in PrCa (Figure 2). In modules M2 and M5, relatively moderate connections were observed. This is likely due to a weak PrCa association (M2, *p*_cor_ = 0.015) or a low preservation score (M5, *Z*_summary_ = 5.2). The biological functions of modules M1 and M2 are associated with the GO_BP term “apoptosis.” Representative genes include *IL6*[60], *SOCS3*[61], *GADD45A*[62], *PIM1*[63], *1L1B*[64], *CDKN1A*[65], *CCL2*[66], *PMAIP1* (also known as *NOXA*) [67], and *RHOB*[68] in module M1, and *DHCR24*[69], *BNIP3*[70], and *IGF1R*[71] in module M2. For modules M3, M4, and M5, although the corresponding GO_BP terms were not directly related to cancer, we found PrCa-relevant genes in these three modules, including *BTG2*[72], *FOS* (also known as *c-Fos*) [73], and *CXCR4*[74] in module M3; *ARFGAP3*[75] and *CDH1*[76] in module M4; and *SMAD3*[77] and *MXI1*[78] in module M5.

Moreover, we identified TF and microRNA regulators enriched in the modules, and the results are shown in Table 3. Some TFs could be seen as associated with several modules. For example, modules M1 and M3 were significantly enriched with the nuclear factor of activated T-cell transcription factor gene, *NFAT* (*p*_M1_ = 3.59 × 10^−7^, *p*_M3_ = 2.20 × 10^−5^). All the other three modules—M2, M4, and M5—were significantly enriched with the gene specificity protein 1, *SP1* (*p*_M2_ = 3.00 × 10^−4^, *p*_M4_ = 4.00 × 10^−4^, *p*_M5_ = 1.20 × 10^−3^) (Figure 2). Both *NFAT* and *SP1* were known to be associated with PrCa [79, 80].

**Table 3:** **Transcription factors (TFs) and microRNAs (miRNAs) identified by the analyses of PrCa-associated modules (M1-M5)**

	No.	Module genes	TF symbol	***p*** ^a^	Module genes	miRNA symbol	***p***
Module M1	1	*CEBPB CXCR4 CTGF PPP1R15A SELE CCL4 EREG SERPINE1 FOS THBD*	*TATA*	1.23 × 10^−5^	*BTG2 GAP43 ADM SMAD7*	hsa-miR-25	2.70 × 10^−3^
	2	*GADD45A CTGF HIF1A GADD45B CCL4 GAP43 SGK1 ERRFI1 FOS IL5 ADM*	*NFAT*[79]	2.20 × 10^−5^	*CEBPB GAP43*	hsa-miR-191	4.00 × 10^−3^
	3	*CEBPB NLRP3 GAP43 EREG HMGB2 ERRFI1 IL5 DUSP1*	*UNKNOWN*	5.19 × 10^−5^	*HMGB2 FOS ADM SMAD7*	hsa-miR-181a	6.10 × 10^−3^
	4	*ATM HIF1A FOS CCL20 EREG*	*TCF1P*	7.40 × 10^−5^	*SGK1 BCL3 CTGF EREG*	*hsa-miR-19a*[82]	6.10 × 10^−3^
	5	*BCL3 CTGF CCL20 GADD45B ERN1*	*CREL*	7.40 × 10^−5^	*ERRFI1 GADD45A GAP43*	hsa-miR-148a	1.19 × 10^−2^
Module M2	1	*DOPEY1 CARTPT RIMS1 CDH1 TLK1 STEAP2 ANP32A TOMM22 CADM1 ADRB2 COG3 XPO7 COPG2 KDELR2 PDIA4*	*UNKNOWN*	6.27 × 10^−5^	*BET1 AP1G1 ARCN1 SEC62*	hsa-miR-409-3p	3.50 × 10^−2^
	2	*TMEM48 SEC62 CADM1 SYNRG AP3M1 DOPEY1 COPZ1 RIMS1 ARFIP1 ANP32A ARFGAP3 GSK3B COG3 TIMM17B LMAN1 AP1G1 SNAPIN*	*SP1*[80]	4.00 × 10^−4^	*RAB14 AP1G1 ATP2C1*	hsa-miR-302b	3.80 × 10^−3^
	3	*SEC23IP ARFIP1 ATP2C1 TLK1 TOMM22 AP3B1 COG3 UHMK1 SYNRG MTX2*	*ELK1*	1.50 × 10^−3^	*RAB14 ARCN1 AP3M1 RERE*	hsa-miR-211	4.70 × 10^−3^
	4	*COPZ1 CARTPT ATP2C1 STEAP2 ANP32A CADM1 UHMK1 AP1G1 SYNRG ARCN1 MTX2 RERE*	*NFAT*	2.60 × 10^−3^	*STRADB CADM1 ADRB2 SYNRG ARCN1 TLK1*	*hsa-miR-15a*[83]	4.70 × 10^−3^
	5	*DOPEY1 CARTPT ERGIC1 AP1M2 CDH1 MIPEP SEC22A ANP32A CADM1 ADRB2 SHROOM2 AP3M1 RPAIN*	*E12*	5.90 × 10^−3^	*BET1 AP1G1 ARCN1 SEC62*	hsa-miR-1	1.14 × 10^−2^
Module M3	1	*SMAD3 KPNA4 KPNA3 LMAN2L REEP1 FLNA PEX14 OPTN TRPS1*	*SP1*	1.20 × 10^−3^	*SMAD3 MXI1 LRP1B KPNA4 TRPS1*	hsa-miR-524	1.00 × 10^−4^
	2	*RTP4 KPNA3 OPTN*	*STAT1*	4.80 × 10^−3^	*SMAD3 NLGN1 LRP1B KPNA4 LMAN2L*	*hsa-miR-15a*	3.00 × 10^−4^
	3	*MXI1 KPNA3 TRPS1*	*CEBPA*	4.80 × 10^−3^	*NLGN1 KPNA4 REEP1 TRPS1*	hsa-miR-493	3.00 × 10^−4^
	4	*SMAD3 BIN3 LMAN2L*	*PITX2*	4.80 × 10^−3^	*MXI1 KPNA3 REEP1*	hsa-miR-24	2.40 × 10^−3^
	5	*GLI3 MXI1 TRPS1*	*CDC5L*	4.80 × 10^−3^	*SMAD3 KPNA3*	hsa-miR-302b	3.70 × 10^−3^
Module M4	1	*CALR EIF5A UNC13B IGF1R TBX3 SOCS2 GLO1 BCL2L1 VEGFA PSEN1*	*SP1*	3.00 × 10^−4^	*BCL2L1 VEGFA DHCR24*	hsa-miR-377	3.00 × 10^−3^
	2	*EIF5A BCL2L1 PPP1R13B SOCS2*	*UNKNOWN*	6.00 × 10^−4^	*PPP2CA SOCS2*	hsa-miR-139	9.00 × 10^−3^
	3	*BCL2L1 VEGFA NME5 BIK*	*RFX1*	6.00 × 10^−4^	*PPP2CA VEGFA CBX4*	*hsa-miR-200b*[84]	9.00 × 10^−3^
	4	*PPP2CA CALR EIF5A PPP1R13B SFN*	*VSX1*	1.90 × 10^−3^	*VEGFA TBX3*	hsa-miR-140	9.00 × 10^−3^
	5	*PPP2CA EIF5A SOCS2*	*RORA*	1.90 × 10^−3^	*PPP2CA VEGFA PPP1R13B*	hsa-miR-29a	9.60 × 10^−3^
Module M5	1	*TNF BTG1 GADD45A CCL2 IL6 GADD45B PIM1 CDKN1A RELA RHOB IER3*	*NFAT*	3.59 × 10^−7^	*GADD45A RHOB SOCS3*	hsa-miR-527	3.50 × 10^−3^
	2	*BCL3 TNFRSF9 TNFSF18 ERN1 GADD45B IER3*	*RELA*	3.59 × 10^−7^	*BTG1 BCL3 RHOB SOCS3*	*hsa-miR-19a*	3.50 × 10^−3^
	3	*BCL3 TNFRSF9 TNFSF18 ERN1 GADD45B IER3*	*REL*	3.59 × 10^−7^	*NLRP3 RHOB*	hsa-miR-223	8.40 × 10^−3^
	4	*TNF BCL3 TNFRSF9 TNFSF18 ERN1 GADD45B*	*NFKB*	3.59 × 10^−7^	*BTG1 PIM1*	hsa-miR-183	2.14 × 10^−2^
	5	*TNFRSF9 GADD45A IL6 PPP1R15A GADD45B CDKN1A PIM1 RELA SERPINB2*	*JUN*	4.86 × 10^−7^	*CUL1 SOCS3*	hsa-miR-203	2.89 × 10^−2^

TFs and miRNAs in italics were those shared between modules and associated with prostate cancer.

^a^ *p* values were adjusted by FDR method.

**Figure 3:**
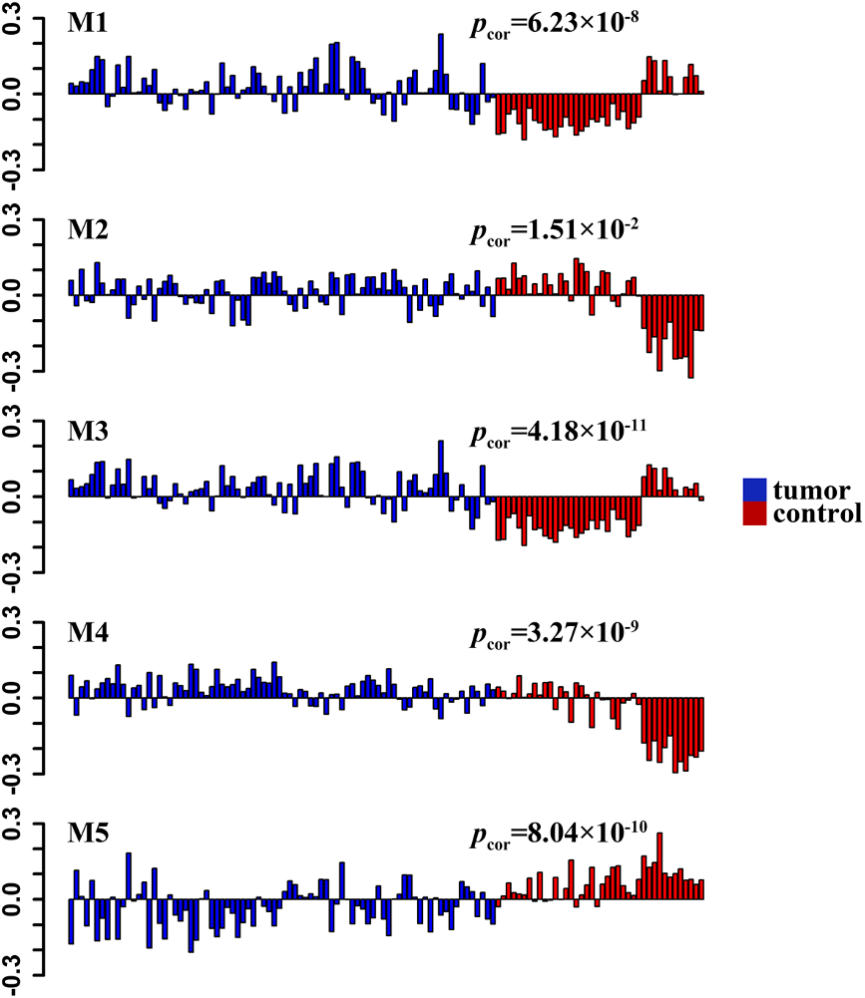
**Expression of module eigengenes in five modules (M1-M5) across samples.** Blue indicates tumor samples, while red indicates control samples.

To further search for evidence of the coordinated regulation of each identified module, we explored the module eigengene in tumor and control samples for all five modules. As shown in Figure 3, the eigengenes of modules M1, M2, M3, and M4 were consistently over-expressed in PrCa tumor samples compared to control samples, while the eigengene of module M5 was under-expressed. These observations implied that the module genes might be co-regulated by the same or similar regulators, e.g., TFs (or their regulators) or miRNAs that regulate the expression of the module genes. We therefore examined the correlation between the candidate TFs (Table 3) with PrCa. Specifically, the TF NFAT family genes, such as *NFATC4* (*p*_cor1_ = 8.94 × 10^−7^, *p*_cor2_ = 7.66 × 10^−4^) and *NFATC1* (*p*_cor1_ = 2.25 × 10^−3^, *p*_cor2_ = 3.61 × 10^−2^), were associated with PrCa. The activation of partner gene *TRPV6* was reported to be critical to NFAT [79, 81] in prostate cancer cells. Our specific examination showed that *TRPV6* was significantly over-expressed in PrCa tumor samples (*p*_cor1_ = 6.49 × 10^−6^) in the training dataset, but not in the testing dataset (*p*_cor2_ = 0.12). In addition, other enriched TFs showed promising evidence (Table 3) for association with PrCa, such as *SP1* (*p*_cor1_ = 4.60 × 10^−7^ and *p*_cor2_ = 7.50 × 10^−5^), *REL* (*p*_cor1_ = 8.08 × 10^-10,^*p*_cor2_ = 0.010), and *JUN* (*p*_cor1_ = 5.91 × 10^-15,^*p*_cor2_ = 7.70 × 10^−5^) in module M1; *RFX1* (*p*_cor1_ = 3.67 × 10^-5,^*p*_cor2_ = 3.10 × 10^−6^), *VSX1* (*p*_cor1_ = 1.55 × 10^-4,^*p*_cor2_ = 0.036), and *RORA* (*p*_cor1_ = 1.29 × 10^-3,^*p*_cor2_ = 3.15 × 10^−11^) in module M2; *NF1* (*p*_cor1_ = 1.14 × 10^-3,^*p*_cor2_ = 6.01 × 10^−5^) in module M4; and *CDC5L* (*p*_cor1_ = 0.012^,^*p*_cor2_ = 8.07 × 10^−6^) in module M5.

In Table 3, we listed the enriched miRNAs; several of them have been reported to be associated with PrCa, such as hsa-miR-19a [82] with modules M1 and M3 (*p*_M1_ = 3.50 × 10^−3^, *p*M3 = 6.10 × 10^−3^), hsa-miR-15a [83] with modules M4 and M5 (*p*_M4_ = 4.70 × 10^−3^, *p*_M5_ = 3.00 × 10^−4^), and hsa-miR-200b [84] with module M2 (*p*_M2_ = 9.00 × 10^−3^). For the other microRNAs, experimental validation is needed to investigate their roles in PrCa.

In order to validate the regulatory TFs/miRNAs detected above, we retrieved RNASeqV2 and miRNASeq data for prostate adenocarcinoma from the TCGA portal to build the expression matrix. The R package edgeR [85] was applied to obtain the differentially expressed genes and miRNAs. The FDR method was applied to adjust *p* values for multiple testing. We found that the identified key TF regulators, including NFAT family genes (*p*_NFATC4_ = 7.85 × 10^−12^, *p*_NFAT5_ = 6.76 × 10^−6^, *p*_NFATC2_ = 6.79 × 10^−6^, *p*_NFATC3_ = 6.95 × 10^−3^), NFAT regulator TRPV6 (*p* = 1.40 × 10^−6^), and SP1 (*p* = 9.49 × 10^−3^), were highly differentially expressed, as well as the other enriched TFs, such as REL (*p* = 1.35 × 10^−5^), RORA (*p* = 1.54 × 10^−13^), and NF1 (*p* = 6.24 × 10^−4^). Similar patterns were also observed in the identified miRNAs, such as hsa-miR-19a (*p* = 1.72 × 10^−12^), hsa-miR-15a (*p* = 8.10 × 10^−10^), and hsa-miR-200b (*p* = 6.39 × 10^−3^).

## Discussion

High-throughput genetic and genomic studies have demonstrated that GO terms are important prior knowledge in facilitating and interpreting of discoveries in complex disease studies. In this study, we identified gene co-expression modules within GO_BP terms for PrCa. We found 118 GO_BP terms that were preserved between training and testing datasets, some of them have been widely studied and reported, such as “programmed cell death” [86], “cell-cell adhesion” [87], and “regulation of apoptosis” [87]. We applied WGCNA to the PrCa expression data sets and identified five co-expression modules which were preserved in the training and testing datasets and enriched with known PrCa genes. In our further evaluation of these modules, we identified several PrCa associated TFs and miRNAs as putative key regulators in PrCa genesis and progression.

To evaluate the performance of our approach, we applied another popular co-expression network reconstruction algorithm, K-means [88], to the GO_BP-based expression matrices. For each GO_BP term, the number of modules obtained from WGCNA was assigned to K-means. Among the 548 constructed modules, only 12 modules showed significant association with PrCa status (FDR < 0.05) and were preserved in the testing dataset (*Z*_summary_ > 5). Further enrichment tests showed that these 12 modules were poorly enriched in the collected PrCa genes (Additional file 3: Table S3). Since the module preservation calculation is computationally time-consuming, we did not perform other algorithms for comparison. Although more comparison with other methods may be needed, the WGCNA approach seems to be effective on detecting the risk modules for PrCa.

The results revealed that the co-expression modules that belong to known cancer-related GO terms could play regulatory roles in PrCa, such as the two apoptosis-related candidate modules, M1 and M2. The results also indicated that those modules associated with general terms, e.g., “response to stress,” “cellular localization,” and “protein localization,” may contribute to PrCa risk in a synergistic way. As a core signaling pathway in cancers [89–92], apoptosis-blocking has proven to be very important in cell development [58, 93] during the stages of progression from normal epithelial cells, to androgen-dependent tumor cells, and further to malignant androgen-independent ones. On the other hand, cells can be activated in various ways in response to stress during cell development, mainly to maintain the balance between cell death and cell proliferation [94]. Therefore, cells that experience too much stress, e.g., an over-expressed module M3, may bring down the rate of cancer cell death and thus result in the formation of cancer [95–97]. As indicated by previous studies [98, 99], we then considered that the alternation of expression for genes involved in cellular and protein localizations play critical roles during cell-division and cancer cell proliferation, such as through modules M4 and M5; studying the localizations of these genes and their encoded proteins can help us elucidate the molecular basis of cancer genesis and progression [100, 101].

As we found, different modules may share the same genetic regulator, such as TF and miRNA. An intriguing example is the TF, NFAT, enriched by both modules M1 and M3. NFAT is reported to promote the epithelial cell proliferation of human primary PrCa [79, 81] with store-independent Ca^2+^ entry via the TRPV6 channel. Significant expression changes of NFAT family genes and their regulator *TRPV6* were observed in PrCa in the datasets. Another TF, SP1, was found to be PrCa-associated and enriched in three modules: M2, M4, and M5. SP1 has been considered an important target for PrCa therapy, since it regulates important genes, like the androgen receptor gene (*AR*), *TGF-β, c-Met* and prostate specific antigen (*PSA*), and others. These genes are involved in cell cycle, proliferation, cell differentiation, and apoptosis [80]. Other enriched TFs, STAT1 and NFKB [102], and moderately enriched miRNAs, hsa-miR-15a [83] and hsa-miR-19a [82] (Table 3), are also reported to be associated with PrCa. Similar expression patterns were also observed in another independent TCGA dataset. Taken together, our findings suggested that these 5 modules and their TF and microRNA regulators are likely critical for the genesis and progression of PrCa. These modules and regulators may be molecular targets for the future development of drugs and new therapies.

In this study, we chose a relatively stringent significance level to detect candidate modules. The identified modules need to be enriched with both eQTL genes and SCNA or mutated genes, with a corrected *p* <0.05. This might exclude moderately associated modules (e.g., *p* <0.2), such as “response to chemical stimulus (in turquoise in Figure 1A4) (*p*_*trans*-eQTL_ = 0.086, *p*_SCNA_ = 0.062),” “regulation of apoptosis (turquoise) (*p*_*cis*-eQTL_ = 0.075, *p*_SCNA_ = 0.039),” and “apoptosis go (turquoise) (*p*_*cis*-eQTL_ = 0.071, *p*_SCNA_ = 0.024)” (Additional file 1: Table S1).

In summary, our findings indicate that genes with same GO functions can cluster into several co-expressed modules to contribute to PrCa, as seen in modules M1 and M2. Modules across GO terms may act in the networks that are regulated by same genetic factors, such as modules M1 and M3. These findings indicate the importance of studying PrCa development at a systems level rather than at a single-gene level, offering insights into the underlying mechanisms of PrCa.

## Conclusions

Using GO_BP terms to start, we conducted gene co-expression analysis of expression profiles of PrCa. Our results revealed five modules that were differentially expressed between tumors and controls, preserved between independent expression datasets, and enriched with putative cancer genes. The enrichment analyses further identified TF and miRNA as key regulators in PrCa. Our study provides important insights for the future investigation of molecular functionality related to PrCa etiology and the development of PrCa diagnosis tools and targeted therapy strategies.

## Electronic supplementary material

Additional file 1: Table S1.: Enrichment analyses of 55 preserved prostate cancer associated modules and preservation summary of corresponding GO_BP terms. (DOCX 47 KB)

Additional file 2: Table S2.: Summarization of modules M1 ~ M5. (DOCX 260 KB)

Additional file 3: Table S3.: Enrichment analyses of 12 preserved prostate cancer-associated modules by K-means algorithm. (DOCX 27 KB)
