# Supplementary material for: Key regulators in prostate cancer identified by co-expression module analysis
Source: BMC Genomics. 2014 Nov 24;15:1015. doi: 10.1186/1471-2164-15-1015 (PMC4258300; doi:10.1186/1471-2164-15-1015)
Supplement: Supplementary file 1 — Additional file 1: Table S1.: Enrichment analyses of 55 preserved prostate cancer associated modules and preservation summary of corresponding GO_BP terms. (DOCX 47 KB) [file 12864_2014_6720_MOESM1_ESM.docx]

**Table S1.** Enrichment analyses of 55 preserved prostate cancer associated modules and preservation summary of corresponding GO_BP terms

| GO_BP term | #Size | *Z*_summary_ | moduleName | *p*_cor_ | #Size | *Z*_summary_ | #enriched cis-eQTL genes | *p_cis_*_-eQTL_ | #enriched trans-eQTL genes | *p_trans_*_-eQTL_ | #enriched SCNA genes | *p*_SCNA_ | #enriched mutation genes | *p*_Mutation_ | #enriched prognistic genes | *p*_prog_ |
| --- | --- | --- | --- | --- | --- | --- | --- | --- | --- | --- | --- | --- | --- | --- | --- | --- |
| Regulation of biological quality | 419 | 9.1 | Regulation of biological quality (blue) | 5.15×10^-8^ | 49 | 11.0 | 14 | 0.67 | 11 | 0.18 | 3 | 0.045 | NA | NA | NA | NA |
|  |  |  | Regulation of biological quality (green) | 3.30×10^-11^ | 30 | 5.9 | 10 | 0.52 | 5 | 0.51 | NA | NA | NA | NA | 4 | 8.03×10^-3^ |
|  |  |  | Regulation of biological quality (red) | 2.21×10^-2^ | 25 | 8.7 | 5 | 0.87 | 7 | 0.096 | 2 | 0.039 | NA | NA | NA | NA |
| Macromolecular complex assembly | 280 | 6.0 | Macromolecular complex assembly (brown) | 3.55×10^-12^ | 45 | 7.4 | 23 | 0.015 | 13 | 0.054 | NA | NA | NA | NA | 2 | 0.21 |
| Biosynthetic process | 470 | 8.9 | Biosynthetic process (blue) | 2.05×10^-5^ | 88 | 8.8 | 47 | 3.43×10^-4^ | 20 | 0.14 | 3 | 0.17 | NA | NA | 6 | 0.059 |
| M phase | 114 | 7.4 | M phase (turquoise) | 2.08×10^-7^ | 35 | 7.1 | 8 | 0.91 | NA | NA | NA | NA | NA | NA | NA | NA |
| Organelle organization and biogenesis | 473 | 6.5 | Organelle organization and biogenesis (brown) | 2.37×10^-5^ | 52 | 7.3 | 24 | 0.040 | 19 | 1.46×10^-3^ | NA | NA | NA | NA | NA | NA |
|  |  |  | Organelle organization and biogenesis (turquoise) | 2.79×10^-12^ | 87 | 5.5 | 28 | 0.62 | 7 | 0.99 | 2 | 0.36 | NA | NA | 6 | 0.061 |
| Anatomical structure morphogenesis | 376 | 14 | Anatomical structure morphogenesis (black) | 8.81×10^-12^ | 15 | 5.8 | 5 | 0.43 | 7 | 6.24×10^-3^ | NA | NA | NA | NA | NA | NA |
|  |  |  | Anatomical structure morphogenesis (blue) | 1.35×10^-3^ | 44 | 6.2 | 17 | 0.28 | 7 | 0.54 | NA | NA | NA | NA | 5 | 0.016 |
|  |  |  | Anatomical structure morphogenesis (turquoise) | 1.31×10^-8^ | 119 | 10.0 | 37 | 0.62 | 33 | 6.28×10^-3^ | NA | NA | NA | NA | NA | NA |
|  |  |  | Anatomical structure morphogenesis (yellow) | 4.16×10^-6^ | 30 | 5.7 | 11 | 0.37 | 5 | 0.48 | NA | NA | NA | NA | NA | NA |
| Defense response | 270 | 15.0 | Defense response (blue) | 1.94×10^-3^ | 63 | 9.6 | 38 | 2.73×10^-4^ | 33 | 2.10×10^-8^ | NA | NA | NA | NA | NA | NA |
| I kappaB kinase nf kappaB cascade | 114 | 5.7 | I kappaB kinase nf kappaB cascade (yellow) | 2.65×10^-5^ | 15 | 5.1 | 6 | 0.34 | 4 | 0.18 | NA | NA | NA | NA | NA | NA |
| Behavior | 153 | 10.0 | Behavior (turquoise) | 1.55×10^-2^ | 44 | 6.2 | 26 | 5.83×10^-3^ | 22 | 3.09×10^-5^ | NA | NA | NA | NA | 4 | 0.11 |
| Cellular biosynthetic process | 321 | 6.2 | Cellular biosynthetic process (blue) | 2.08×10^-6^ | 43 | 7.1 | 27 | 2.73×10^-4^ | 9 | 0.26 | NA | NA | NA | NA | 2 | 0.20 |
| Cell cell signaling | 404 | 5.5 | Cell cell signaling (blue) | 3.34×10^-11^ | 83 | 8.1 | 25 | 0.63 | 17 | 0.22 | 4 | 0.057 | NA | NA | 2 | 0.54 |
|  |  |  | Cell cell signaling (brown) | 2.88×10^-4^ | 49 | 8.4 | 20 | 0.16 | 17 | 4.60×10^-3^ | 2 | 0.12 | NA | NA | NA | NA |
| Inflammatory response | 129 | 7.7 | Inflammatory response (turquoise) | 1.95×10^-3^ | 34 | 6.1 | 22 | 1.45×10^-3^ | 18 | 3.09×10^-5^ | NA | NA | NA | NA | NA | NA |
| Response to stress | 508 | 10.0 | Response to stress (green) | 4.18×10^-11^ | 37 | 8.3 | 19 | 0.017 | 14 | 3.16×10^-3^ | NA | NA | 2 | 1.37×10^-3^ | NA | NA |
|  |  |  | Response to stress (pink) | 1.25×10^-12^ | 26 | 6.4 | 13 | 0.077 | 8 | 0.086 | NA | NA | NA | NA | 2 | 0.091 |
|  |  |  | Response to stress (red) | 4.42×10^-7^ | 33 | 11.0 | 12 | 0.41 | 7 | 0.28 | NA | NA | NA | NA | 2 | 0.14 |
|  |  |  | Response to stress (turquoise) | 1.90×10^-4^ | 72 | 6.9 | 24 | 0.47 | 18 | 0.086 | NA | NA | NA | NA | 4 | 0.13 |
| Response to chemical stimulus | 314 | 15.0 | Response to chemical stimulus (green) | 5.29×10^-3^ | 30 | 7.1 | 11 | 0.43 | 8 | 0.15 | NA | NA | NA | NA | 2 | 0.12 |
|  |  |  | Response to chemical stimulus (red) | 3.79×10^-3^ | 28 | 5.8 | 18 | 3.07×10^-3^ | 9 | 0.063 | NA | NA | NA | NA | 4 | 0.14 |
|  |  |  | Response to chemical stimulus (turquoise) | 3.72×10^-10^ | 59 | 7.6 | 22 | 0.28 | 15 | 0.086 | 3 | 0.062 | NA | NA | NA | NA |
| Post translational protein modification | 476 | 7.2 | Post translational protein modification (turquoise) | 3.64×10^-4^ | 70 | 5.5 | 22 | 0.67 | 8 | 0.93 | NA | NA | NA | NA | 4 | 0.13 |
|  |  |  | Post translational protein modification (yellow) | 3.95×10^-9^ | 40 | 5.7 | 12 | 0.69 | 12 | 0.063 | 2 | 0.090 | NA | NA | 2 | 0.19 |
| Cellular component assembly | 298 | 5.6 | Cellular component assembly (blue) | 2.65×10^-11^ | 50 | 7.4 | 23 | 0.043 | 13 | 0.087 | NA | NA | NA | NA | 2 | 0.26 |
| Proteolysis | 191 | 5.7 | Proteolysis (turquoise) | 7.55×10^-8^ | 46 | 5.1 | 20 | 0.087 | 10 | 0.22 | NA | NA | NA | NA | 2 | 0.22 |
| Response to biotic stimulus | 120 | 8.1 | Response to biotic stimulus (blue) | 2.35×10^-5^ | 27 | 5.0 | 19 | 2.73×10^-4^ | 12 | 1.18×10^-3^ | NA | NA | NA | NA | NA | NA |
| Macromolecule biosynthetic process | 321 | 5.1 | Macromolecule biosynthetic process (blue) | 3.00×10^-7^ | 50 | 5.8 | 22 | 0.084 | 13 | 0.096 | 3 | 0.051 | NA | NA | 8 | 1.62×10^-3^ |
| Secretion | 178 | 10.0 | Secretion (turquoise) | 2.19×10^-6^ | 42 | 6.4 | 19 | 0.060 | 8 | 0.31 | NA | NA | NA | NA | 5 | 0.013 |
| Protein complex assembly | 167 | 7.5 | Protein complex assembly (turquoise) | 4.61×10^-8^ | 44 | 7.5 | 22 | 0.021 | 13 | 0.047 | NA | NA | NA | NA | NA | NA |
| Immune system process | 332 | 18.0 | Immune system process (green) | 1.63×10^-8^ | 20 | 6.1 | 13 | 3.89×10^-3^ | 11 | 2.44×10^-4^ | NA | NA | NA | NA | NA | NA |
| Response to external stimulus | 312 | 13.0 | Response to external stimulus (green) | 1.42×10^-8^ | 27 | 8.2 | 9 | 0.43 | 9 | 0.035 | NA | NA | NA | NA | NA | NA |
|  |  |  | Response to external stimulus (red) | 1.19×10^-8^ | 26 | 5.1 | 10 | 0.34 | 7 | 0.13 | 3 | 0.024 | NA | NA | 2 | 0.086 |
| Cellular localization | 371 | 12.0 | Cellular localization (green) | 4.48×10^-8^ | 28 | 5.7 | 14 | 0.071 | 8 | 0.10 | NA | NA | NA | NA | NA | NA |
|  |  |  | Cellular localization (turquoise) | 3.27×10-9 | 70 | 5.7 | 35 | 7.30×10^-3^ | 9 | 0.85 | 6 | 0.024 | NA | NA | 10 | 1.62×10^-3^ |
| Mitosis | 82 | 7.1 | Mitosis (turquoise) | 1.15×10^-7^ | 29 | 5.9 | 7 | 0.83 | NA | NA | NA | NA | NA | NA | NA | NA |
| Cytoskeleton organization and biogenesis | 208 | 7.8 | Cytoskeleton organization and biogenesis (turquoise) | 2.09×10^-5^ | 46 | 6.4 | 26 | 2.16×10^-3^ | 18 | 1.08×10^-3^ | NA | NA | NA | NA | NA | NA |
| Response to wounding | 190 | 9.6 | Response to wounding (blue) | 1.00×10^-5^ | 35 | 6.5 | 19 | 0.017 | 15 | 1.18×10^-3^ | NA | NA | NA | NA | NA | NA |
| Phosphorylation | 313 | 5.5 | Phosphorylation (black) | 4.96×10^-7^ | 18 | 5.7 | 6 | 0.48 | 6 | 0.077 | NA | NA | 2 | 4.93×10^-4^ | NA | NA |
| Protein localization | 214 | 7.8 | Protein localization (brown) | 8.04×10^-10^ | 24 | 5.2 | 13 | 0.034 | 5 | 0.29 | 2 | 0.039 | 2 | 6.84×10^-4^ | NA | NA |
| Regulation of multicellular organismal process | 151 | 8.7 | Regulation of multicellular organismal process (blue) | 1.24×10^-11^ | 28 | 7.3 | 10 | 0.41 | 6 | 0.26 | NA | NA | NA | NA | NA | NA |
| Regulation of apoptosis | 341 | 6.4 | Regulation of apoptosis (green) | 1.51×10^-2^ | 26 | 6.1 | 15 | 8.93×10^-3^ | 7 | 0.11 | 2 | 0.040 | NA | NA | 2 | 0.081 |
|  |  |  | Regulation of apoptosis (red) | 1.32×10^-7^ | 22 | 8.1 | 12 | 0.027 | 9 | 6.59×10^-3^ | NA | NA | 3 | 6.39×10^-5^ | NA | NA |
|  |  |  | Regulation of apoptosis (turquoise) | 8.71×10^-7^ | 60 | 5.5 | 26 | 0.075 | 12 | 0.29 | 4 | 0.039 | NA | NA | 2 | 0.36 |
| Apoptosis go | 431 | 7.3 | Apoptosis go (black) | 2.55×10^-8^ | 26 | 11.0 | 15 | 8.93×10^-3^ | 12 | 1.08×10^-3^ | NA | NA | 3 | 6.41×10^-5^ | NA | NA |
|  |  |  | Apoptosis go (turquoise) | 4.90×10^-6^ | 75 | 5.0 | 32 | 0.071 | 16 | 0.22 | 6 | 0.024 | NA | NA | 2 | 0.50 |
| M phase of mitotic cell cycle | 85 | 8.4 | M phase of mitotic cell cycle (turquoise) | 1.21×10^-7^ | 30 | 6.6 | 7 | 0.85 | NA | NA | NA | NA | NA | NA | NA | NA |
| Reproduction | 265 | 5.4 | Reproduction (green) | 5.57×10^-5^ | 13 | 5.2 | 4 | 0.48 | 3 | 0.22 | NA | NA | NA | NA | NA | NA |
|  |  |  | Reproduction (yellow) | 3.23×10^-3^ | 31 | 6.4 | 12 | 0.28 | 7 | 0.21 | NA | NA | NA | NA | 2 | 0.11 |
| Regulation of cell proliferation | 308 | 5.8 | Regulation of cell proliferation (blue) | 6.26×10^-7^ | 41 | 6.0 | 14 | 0.43 | 10 | 0.14 | NA | NA | NA | NA | NA | NA |
|  |  |  | Regulation of cell proliferation (red) | 2.25×10^-6^ | 23 | 9.7 | 9 | 0.28 | 7 | 0.077 | NA | NA | NA | NA | NA | NA |
