# Supplementary material for: Key regulators in prostate cancer identified by co-expression module analysis
Source: BMC Genomics. 2014 Nov 24;15:1015. doi: 10.1186/1471-2164-15-1015 (PMC4258300; doi:10.1186/1471-2164-15-1015)
Supplement: Supplementary file 2 — Additional file 2: Table S2.: Summarization of modules M1 ~ M5. (DOCX 260 KB) [file 12864_2014_6720_MOESM2_ESM.docx]

**Table S2.** Summarization of modules M1~M5

| Genes | kME | *cis*-eQTL | *trans*-eQTL | CNV | Mutation | Symbol |
| --- | --- | --- | --- | --- | --- | --- |
| M1 | | | | | | |
| *SERPINE1* | 0.871 | √ | √ |  |  | 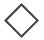 |
| *PPP1R15A* | 0.869 | √ |  |  |  | 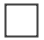 |
| *GADD45A* | 0.862 | √ | √ |  |  | 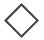 |
| *BTG2* | 0.858 | √ |  |  |  | 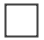 |
| *CCL4* | 0.847 | √ | √ |  |  | 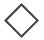 |
| *GADD45B* | 0.843 | √ | √ |  |  | 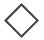 |
| *SELE* | 0.841 |  |  |  |  | 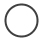 |
| *FOS* | 0.840 |  |  |  |  | 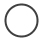 |
| *CXCR4* | 0.823 | √ | √ |  |  | 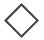 |
| *IL8* | 0.821 |  |  |  |  | 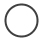 |
| *CEBPB* | 0.819 | √ |  |  |  | 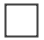 |
| *CXCL2* | 0.816 |  |  |  |  | 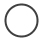 |
| *DUSP1* | 0.814 |  |  |  |  | 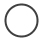 |
| *THBD* | 0.788 |  |  |  |  | 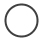 |
| *CTGF* | 0.780 |  |  |  |  | 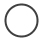 |
| *SGK1* | 0.775 | √ | √ |  |  | 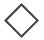 |
| *ERRFI1* | 0.730 |  |  |  |  | 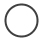 |
| *NLRP3* | 0.673 | √ | √ |  |  | 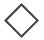 |
| *ADM* | 0.650 |  |  |  |  | 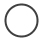 |
| *BCL3* | 0.629 |  |  |  |  | 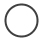 |
| *SMAD7* | 0.598 | √ |  |  |  | 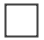 |
| *HMGB2* | 0.595 | √ |  |  |  | 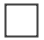 |
| *ERN1* | 0.572 |  |  |  |  | 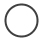 |
| *EREG* | 0.566 |  |  |  |  | 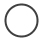 |
| *HIF1A* | 0.537 |  |  |  |  | 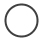 |
| *LYZ* | 0.486 |  |  |  |  | 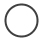 |
| *CCL20* | 0.485 | √ | √ |  |  | 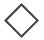 |
| *ELF3* | 0.482 | √ | √ |  |  | 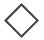 |
| *DDIT3* | 0.466 |  |  |  |  | 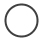 |
| *CHEK2* | 0.436 | √ |  |  | √ | 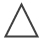 |
| *GAP43* | 0.436 |  |  |  |  | 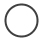 |
| *C4BPB* | 0.428 |  |  |  |  | 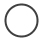 |
| *KLRG1* | 0.390 | √ | √ |  |  | 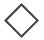 |
| *IL5* | 0.357 |  |  |  |  | 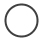 |
| *PROS1* | 0.356 |  |  |  |  | 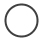 |
| *ACHE* | 0.342 | √ |  |  |  | 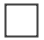 |
| *ATM* | 0.334 |  |  |  | √ | 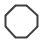 |
| M2 | | | | | | |
| *DOPEY2* | 0.885 | √ | √ |  |  | 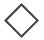 |
| *GOLGA5* | 0.868 |  |  |  |  | 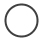 |
| *ARFGAP3* | 0.860 | √ |  |  |  | 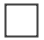 |
| *STEAP2* | 0.847 | √ | √ |  |  | 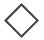 |
| *AP1M2* | 0.828 |  |  |  |  | 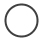 |
| *COG3* | 0.826 |  |  | √ |  | 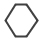 |
| *MTX2* | 0.825 | √ |  |  |  | 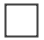 |
| *SEC23IP* | 0.825 |  |  |  |  | 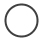 |
| *AP3M1* | 0.805 |  |  |  |  | 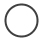 |
| *ZWINT* | 0.799 |  |  |  |  | 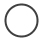 |
| *CDH1* | 0.791 | √ | √ |  |  | 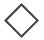 |
| *HPS4* | 0.785 |  |  |  |  | 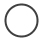 |
| *GOSR1* | 0.770 |  |  |  |  | 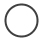 |
| *BET1* | 0.766 | √ |  |  |  | 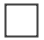 |
| *SLC25A15* | 0.764 | √ |  |  |  | 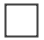 |
| *COX18* | 0.752 |  |  |  |  | 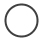 |
| *ANP32A* | 0.751 |  |  |  |  | 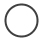 |
| *PEX7* | 0.744 |  |  |  |  | 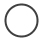 |
| *KDELR2* | 0.719 |  |  |  |  | 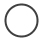 |
| *LMAN1* | 0.714 |  |  |  |  | 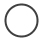 |
| *ARFIP1* | 0.713 |  |  |  |  | 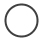 |
| *KRT18* | 0.712 | √ | √ |  |  | 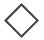 |
| *PDIA4* | 0.709 | √ | √ |  |  | 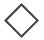 |
| *AP3B1* | 0.708 | √ |  |  |  | 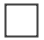 |
| *STRADB* | 0.707 |  |  |  |  | 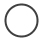 |
| *ATP2C1* | 0.703 | √ |  |  |  | 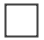 |
| *ADRB2* | 0.682 | √ |  |  |  | 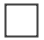 |
| *RAB14* | 0.678 |  |  |  |  | 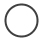 |
| *MIPEP* | 0.673 |  |  |  |  | 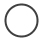 |
| *COPB2* | 0.666 | √ |  |  |  | 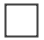 |
| *ARCN1* | 0.660 |  |  |  |  | 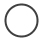 |
| *GSK3B* | 0.656 |  |  |  |  | 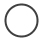 |
| *ERGIC1* | 0.648 |  |  |  |  | 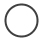 |
| *COPZ1* | 0.636 |  |  |  |  | 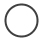 |
| *CADM1* | 0.632 | √ | √ |  |  | 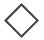 |
| *DUSP16* | 0.621 | √ |  | √ |  | 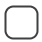 |
| *CSPG5* | 0.612 |  |  |  |  | 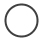 |
| *RIMS1* | 0.595 |  |  |  |  | 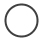 |
| *PEX19* | 0.586 | √ |  |  |  | 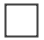 |
| *TOMM22* | 0.582 |  |  |  |  | 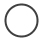 |
| *SHROOM2* | 0.574 |  |  |  |  | 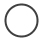 |
| *PEX3* | 0.530 | √ |  |  |  | 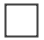 |
| *SYNRG* | 0.485 |  |  |  |  | 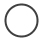 |
| *CARTPT* | 0.346 | √ |  |  |  | 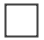 |
| M3 | | | | | | |
| *TRIP6* | 0.802 | √ | √ |  |  | 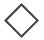 |
| *FLNA* | 0.797 |  |  |  |  | 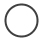 |
| *MAL* | 0.793 |  |  |  |  | 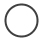 |
| *SMAD3* | 0.780 | √ |  |  |  | 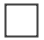 |
| *OPTN* | 0.754 |  |  |  |  | 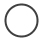 |
| *PEX14* | 0.731 | √ |  |  |  | 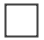 |
| *KPNA3* | 0.672 | √ |  |  |  | 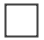 |
| *LRP1B* | 0.666 |  |  | √ | √ | 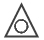 |
| *CD81* | 0.652 | √ |  |  |  | 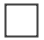 |
| *BIN3* | 0.613 | √ |  | √ |  | 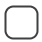 |
| *KPNA4* | 0.602 |  |  |  |  | 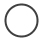 |
| *GLI3* | 0.574 |  |  |  |  | 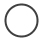 |
| *MXI1* | 0.563 |  |  |  |  | 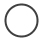 |
| *KLHL2* | 0.551 |  |  |  |  | 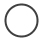 |
| *TRPS1* | 0.547 | √ |  |  |  | 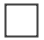 |
| *NOD2* | 0.539 | √ |  |  |  | 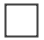 |
| *PYCARD* | 0.537 | √ |  |  |  | 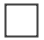 |
| *ARL4D* | 0.525 |  |  |  |  | 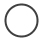 |
| *NLGN1* | 0.523 |  |  |  |  | 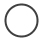 |
| *REEP1* | 0.523 | √ | √ |  |  |  |
| *TAF3* | 0.514 | √ |  |  |  |  |
| *LMAN2L* | 0.494 |  |  |  |  |  |
| *RTP4* | 0.410 | √ | √ |  |  |  |
| *SQSTM1* | 0.311 | √ |  |  |  |  |
| *RTP1* | 0.307 |  |  |  |  |  |
| M4 | | | | | | |
| *DHCR24* | 0.795 | √ |  |  |  |  |
| *UNC13B* | 0.773 | √ | √ |  |  |  |
| *ALOX15B* | 0.770 |  |  |  |  |  |
| *CD38* | 0.752 | √ | √ |  |  |  |
| *BNIP3* | 0.734 |  |  |  |  |  |
| *PSEN1* | 0.724 |  |  |  |  |  |
| *MOAP1* | 0.711 | √ | √ |  |  |  |
| *IGF1R* | 0.704 |  |  |  |  |  |
| *SOCS2* | 0.688 | √ | √ |  |  |  |
| *PPP1R13B* | 0.687 |  |  |  |  |  |
| *BIK* | 0.676 | √ | √ |  |  |  |
| *NME5* | 0.625 |  |  | √ |  |  |
| *GCLC* | 0.620 | √ |  |  |  |  |
| *CALR* | 0.569 | √ |  |  |  |  |
| *CBX4* | 0.567 |  |  |  |  |  |
| *SFN* | 0.551 | √ | √ |  |  |  |
| *PLG* | 0.547 | √ |  |  |  |  |
| *GLO1* | 0.531 | √ |  |  |  |  |
| *MAPK8* | 0.488 |  |  |  |  |  |
| *SCIN* | 0.475 | √ |  |  |  |  |
| *VEGFA* | 0.468 | √ |  |  |  |  |
| *TBX3* | 0.436 |  |  |  |  |  |
| *EIF5A* | 0.426 | √ |  |  |  |  |
| *PPP2CA* | 0.412 |  |  | √ |  |  |
| *HBXIP* | 0.403 | √ |  |  |  |  |
| *BCL2L1* | 0.347 |  |  |  |  |  |
| M5 | | | | | | |
| *IL6* | 0.924 | √ | √ |  |  |  |
| *SOCS3* | 0.909 |  |  |  |  |  |
| *PPP1R15A* | 0.882 | √ |  |  |  |  |
| *GADD45A* | 0.880 | √ | √ |  |  |  |
| *IER3* | 0.879 | √ | √ |  |  |  |
| *GADD45B* | 0.864 | √ | √ |  |  |  |
| *MCL1* | 0.859 | √ |  |  |  |  |
| *PIM1* | 0.835 | √ | √ |  | √ |  |
| *BCL2A1* | 0.825 | √ | √ |  |  |  |
| *IL1B* | 0.825 | √ | √ |  |  |  |
| *CDKN1A* | 0.806 |  | √ |  | √ |  |
| *CCL2* | 0.801 |  |  |  |  |  |
| *CLCF1* | 0.793 | √ | √ |  |  |  |
| *TNFSF9* | 0.753 | √ |  |  |  |  |
| *PMAIP1* | 0.738 |  |  |  |  |  |
| *RHOB* | 0.729 | √ | √ |  |  |  |
| *BCL3* | 0.702 |  |  |  |  |  |
| *STK17A* | 0.640 |  |  |  |  |  |
| *NLRP3* | 0.618 | √ | √ |  |  |  |
| *BTG1* | 0.574 |  |  |  |  |  |
| *ERN1* | 0.538 |  |  |  |  |  |
| *APOE* | 0.508 | √ |  |  |  |  |
| *TNFRSF9* | 0.505 |  |  |  |  |  |
| *CUL1* | 0.496 |  |  |  |  |  |
| *CHEK2* | 0.433 | √ |  |  | √ |  |
| *RELA* | 0.381 |  |  |  |  |  |
| *TNF* | 0.370 | √ | √ |  |  |  |
| *TNFSF18* | 0.336 |  |  |  |  |  |
| *SERPINB2* | 0.312 | √ | √ |  |  |  |
