# Supplementary material for: Key regulators in prostate cancer identified by co-expression module analysis
Source: BMC Genomics. 2014 Nov 24;15:1015. doi: 10.1186/1471-2164-15-1015 (PMC4258300; doi:10.1186/1471-2164-15-1015)
Supplement: Supplementary file 3 — Additional file 3: Table S3.: Enrichment analyses of 12 preserved prostate cancer-associated modules by K-means algorithm. (DOCX 27 KB) [file 12864_2014_6720_MOESM3_ESM.docx]

**Table S3.** Enrichment analyses of 12 preserved prostate cancer associated modules by K-means algorithm

| GO_BP term | #Size | *Z*_summary_ | moduleName | *p*_cor_ | #Size | *Z*_summary_ | #enriched cis-eQTL genes | *p_cis_*_-eQTL_ | #enriched trans-eQTL genes | *p_trans_*_-eQTL_ | #enriched SCNA genes | *p*_SCNA_ | #enriched mutation genes | *p*_Mutation_ | #enriched prognistic genes | *p*_prog_ |
| --- | --- | --- | --- | --- | --- | --- | --- | --- | --- | --- | --- | --- | --- | --- | --- | --- |
| Muscle development | 93 | 9.8 | Macromolecular complex assembly (2) | 3.61×10^-3^ | 14 | 8.3 | 5 | 0.61 | 2 | 0.61 | NA | NA | NA | NA | NA | NA |
| Intracellular protein transport | 145 | 8.1 | Intracellular protein transport (5) | 2.43×10^-3^ | 19 | 5.2 | 6 | 0.69 | 3 | 0.59 | NA | NA | NA | NA | 2 | 0.15 |
|  |  |  | Intracellular protein transport (6) | 4.73×10^-4^ | 16 | 7.4 | 7 | 0.39 | 4 | 0.33 | NA | NA | NA | NA | NA | NA |
| Locomotory behavior | 95 | 9.4 | Locomotory behavior (4) | 1.10×10^-3^ | 32 | 7.8 | NA | NA | NA | NA | 2 | 0.037 | NA | NA | NA | NA |
| Regulation of developmental process | 440 | 8.3 | Regulation of developmental process (6) | 4.08×10^-3^ | 27 | 9.9 | NA | NA | NA | NA | NA | NA | 3 | 1.7×10^-5^ | NA | NA |
| Alcohol metabolic process | 88 | 7.7 | Alcohol metabolic process (1) | 6.79×10^-13^ | 25 | 8.4 | NA | NA | 7 | 0.23 | NA | NA | NA | NA | 4 | 0.016 |
| Cellular homeostasis | 147 | 8 | Cellular homeostasis (6) | 1.52×10^-2^ | 16 | 7.4 | 9 | 0.11 | 9 | 5.36×10^-3^ | 2 | 0.014 | NA | NA | NA | NA |
| Response to external stimulus | 312 | 13 | Response to external stimulus (9) | 6.71×10^-8^ | 28 | 8.8 | NA | NA | NA | NA | NA | NA | NA | NA | NA | NA |
| Cellular localization | 371 | 12 | Cellular localization (4) | 4.06×10^-4^ | 40 | 7.8 | NA | NA | 7 | 0.55 | NA | NA | NA | NA | NA | NA |
| Ion homeostasis | 129 | 7.5 | Ion homeostasis (2) | 3.02×10^-12^ | 19 | 8.6 | NA | NA | NA | NA | 2 | 0.14 | NA | NA | NA | NA |
| Negative regulation of developmental process | 197 | 6.6 | Negative regulation of developmental process (3) | 4.03×10^-3^ | 15 | 8 | NA | NA | 6 | 0.10 | NA | NA | NA | NA | NA | NA |
| Ectoderm development | 80 | 5.6 | Ectoderm development (1) | 3.61×10^-10^ | 11 | 7 | 3 | 0.77 | 3 | 0.31 | NA | NA | NA | NA | NA | NA |
